# Developments in diagnostic and surgical techniques in children with sagittal suture craniosynostosis: a systematic review spanning the last 30 years

**DOI:** 10.1186/s13023-025-03978-9

**Published:** 2025-08-17

**Authors:** Julia Hermann, Christa K. Raak, Thomas Ostermann, Wolfram Scharbrodt

**Affiliations:** 1 Integrative Neuromedicine, Community Hospital Herdecke, Witten/Herdecke University, Gerhard-Kienle-Weg 4, 58313 Herdecke, Germany; 2 Center for Integrative Medicine, Faculty of Health, School of Medicine, Witten/Herdecke University, Alfred-Herrhausen-Straße 50, 58458 Witten, Germany; 3 Department of Psychology and Psychotherapy, Witten/Herdecke University, Alfred-Herrhausen-Straße 50, 58458 Witten, Germany

**Keywords:** Development of surgical interventions, Sagittal suture craniosynostosis, Literature review, Rare diseases

## Abstract

**Background:**

Sagittal suture craniosynostosis is the most usual subtype of craniosynostosis which results from premature fusion of the sagittal suture. It leads to an elongated skull shape known as scaphocephaly. This condition necessitates timely surgical intervention to correct cranial deformities and prevent the associated complications. Over the past three decades, the use of advanced diagnostic methods and the refinement of surgical techniques have improved the understanding of this rare disease.

**Objective:**

To analyse the development of surgical interventions and diagnostic methods in children suffering from sagittal suture craniosynostosis over the last three decades.

**Methods:**

A comprehensive literature search was conducted in electronic databases Pubmed and online university libraries to identify articles, studies and case reports reporting on surgical interventions and diagnostic procedures for sagittal suture craniosynostosis the period from 1994 to 2024. Clinical studies, case reports, systematic reviews and meta-analyses were assessed and analysed according to inclusion and exclusion criteria. Prisma guidelines for systematic reviews were considered.

**Results:**

A systematic literature search identified 301, and a hand search identified 12 articles, of which a total of 57 met the inclusion criteria after careful evaluation. The reviewed studies, predominantly originated from the USA and the Netherlands and provided data on diagnostic methods, surgical techniques, patient-specific characteristics, and outcomes for non-syndromic sagittal craniosynostosis.

**Conclusions:**

The evolutionary change in surgical and diagnostic strategies for sagittal suture craniosynostosis reflects the ongoing efforts of the medical community to achieve optimal outcomes for affected children. The selection of the appropriate technique remains an individualized decision, considering age, severity of craniosynostosis and other patient-specific factors.

## Introduction

Craniosynostosis (CSO) is a rare congenital disorder characterized by the premature fusion of cranial sutures, which result in abnormal skull development and, in severe cases, lead to cognitive impairments due to restricted brain growth. The most common subtype, sagittal suture craniosynostosis, involves the early ossification of the sagittal suture, causing an elongated head shape known as scaphocephaly [1–3].

From an epidemiological perspective, the incidence of a child born with craniosynostosis is approximately one in 2500 live births [4, 5]. Isolated, non-syndromic craniosynostosis has an incidence of 6 cases per 100,000 live births, with isolated sagittal suture synostosis being the most frequent subtype [2, 5].

In recent decades, advancements in diagnostic imaging and surgical techniques have significantly improved the early detection and management of this condition, enabling better clinical outcomes. Surgical approaches have evolved from simple linear craniectomies to more complex cranial vault reconstructions (CVR) as well as minimal invasive approaches, highlighting continued efforts to refine treatment approaches for this complex condition [1, 6].

The optimal strategy for diagnosing and managing craniosynostosis remains a subject of ongoing controversy among medical experts. The diagnosis of craniosynostosis is typically based on a clinical examination focused on skull shape. Additional methods, such as head circumference measurement, cranial ultrasound (CUS), and 3D stereophotography, may be used, with computed tomography (CT) or magnet resonance imaging (MRI) reserved for complex cases [2, 7, 8]. Treatment involves two main approaches: endoscopic-assisted surgery and open surgery. The endoscopic method offers benefits like reduced blood loss, shorter operation times, and minimal scarring but often requires postoperative helmet therapy. Open surgery provides immediate correction but is more invasive, with higher risks of complications like significant blood loss and scarring [9–11].

Endoscopic surgery demonstrates a significantly lower mortality rate compared to open techniques, attributed to its minimally invasive nature and reduced surgical trauma. While both methods have low overall mortality, the open approach carries a slightly higher risk due to prolonged operative times and greater surgical burden. Endoscopic procedures are particularly advantageous when performed early in life, as they involve smaller incisions and facilitate quicker recovery [12–15].

Controversies persist regarding the optimal treatment, including surgical techniques, ideal surgical age and long-term developmental outcomes.

Depending on the cause, craniosynostosis can occur in isolation or non-syndromic, but also as part of syndromic genetic diseases, such as Apert, Crouzon, Muenke or Saethre-Chotzen syndrome, to name a few [3, 4, 16]. In 20–30% of cases, primary multisutural craniosynostosis is observed in the context of complex syndromic diseases, which usually follow an autosomal dominant transmission pattern [4, 17]. These syndromes are increasingly caused by autosomal-dominantly caused mutations in genes that influence cartilage and bone growth, such as mutations in the fibroblast growth factor gene 1–3 on chromosomes 4, 8, 10 and the TWIST 1 gene on chromosome 7 [4, 16, 18]. The mutated genes of fibroblast growth factor 1–3 apparently play a prominent role [2, 18]. However, the majority of all craniosynostoses occur in isolation, irregularly and with an ambiguous aetiology [3, 17].

A genetic cause can also be assumed for non-syndromic craniosynostosis, as for example the ratio of male to female is 3:1 to 4:1, a familial clustering of 2% has been described [19] and in twin pairs both children are most likely to be affected [5, 20]. The incidence of single suture synostosis increased noticeable constant in recent years. Moreover, there is a dynamic change in the frequency of the different subtypes of this clinical picture at the same time [21–24].

Although this article primarily focuses on sagittal craniosynostosis, the environmental and metabolic factors discussed herein broadly pertain to isolated craniosynostoses. The literature increasingly discusses following factors like maternal smoking, trauma or infections during pregnancy, and advanced paternal age [17]. Disorders of bone metabolism that indirectly affect skull growth—such as hypophosphatemia, rickets, mucopolysaccharidoses, and hyperthyroidism—have been identified as potential contributing factors [4, 16]. Haematological conditions including thalassemia, sickle cell anaemia, and polycythaemia vera have also been described in that context [16]. A lack of sufficient intracranial growth pressure during development, for instance due to microcephaly associated with cerebral malformations [4], may promote the onset of craniosynostosis [16]. Intrauterine compression of suture regions, caused by uterine deformities or tumours, has likewise been reported [16]. Prenatal exposure to the anticonvulsant valproate is also considered a potential trigger [16]. In rare cases, craniosynostosis may occur following the drainage of hydrocephalus, particularly due to shunt over drainage [3, 16].

The aetiologic classification of craniosynostosis is challenging due to the large number of related genes. As an option, classification based on anatomical characteristics has proven successful. Depending on the location of the affected suture and the number of simultaneously affected sutures, a recurring appearance develops [16].

Ossification of the sagittal suture leads to a longitudinal skull, known as scaphocephaly [4, 16]. This shows a characteristic skull deformity, characterised by a reduced extension of the bitemporal dimension and a compensatory lengthening of the skull in the anterior–posterior direction. A characteristic prominence of the forehead and occipital region develops, commonly referred to as "frontal and occipital bossing" [5]. In addition, the abnormal characteristic head shape, the occlusion of usually several sutures and consecutive disproportion between brain growth and skull size can result in organic complications, including increased intracranial pressure (ICP) and developmental disorders [3, 4].

Early diagnosis is essential, as timely intervention can prevent a pronounced deformity, potential complications, such as ICP or developmental delays. Furthermore, advancements in minimally invasive surgical techniques allow for improved outcomes, including quicker recovery times and reduced risks. Familiarity with this condition enables healthcare professionals to provide optimal care, ensuring better long-term prognosis for affected children​.

This review aims to analyse the development of surgical interventions and diagnostic methods related to sagittal suture craniosynostosis in children over the last three decades. It is to highlight milestones, challenges and prospects in this specialised field of paediatric neurosurgery.

## Methods

This review was performed according to the PRISMA Guidelines for Systematic Reviews and Meta-Analyses [25]. Although they refer to randomized trials they can also be used as a basis for reporting systematic reviews of other types of research, particularly for the evaluation of interventions. As this review focuses on diagnostics and surgical techniques, we thus followed PRISMA Guidelines whenever possible.

### Search methods

We carried out systematic literature research through the electronic databases Medline/PubMed and the medical libraries Ruhr University Bochum and Witten Herdecke University from 1994 until 2024. The methodological approach involved the use of a total of two search strings, with the first search string targeting therapeutic or surgical perspectives and the second focusing on diagnostic aspects or technological advances and their outcomes.

Search 1: (((craniosynostosis [Title/Abstract]) AND ((monosutural) OR (single suture))) AND ((sagittal) OR (sagittal suture))) AND ((((operative technique) OR (surgical methods)) OR (surgical innovations)) OR (surgical procedures)) Filters: from 1994/1/1 - 2024/6/1

Search 2: (((craniosynostosis [Title/Abstract]) AND ((monosutural) OR (single suture))) AND ((sagittal) OR (sagittal suture))) AND ((diagnostics) OR (procedures)) Filters: from 1994/1/1 - 2024/6/1

### Study eligibility criteria

Articles were initially selected based on an initial screening of the titles and abstracts using predefined inclusion and exclusion criteria for both search strings.

Inclusion criteria for the literature review were:

- Clinical studies, case reports, systematic reviews, and meta-analyses published between 1993 and 2024 in English or German.
- Studies were required to focus on the diagnosis and surgical techniques of non-syndromic sagittal craniosynostosis.

Articles were excluded if they reported:

- Studies that were not directly related to the specific diagnostic and surgical aspects of a sagittal craniosynostosis
- Studies that consisted solely of surveys asking parents or surgeons about subjective assessments of cosmetic outcomes after surgery, as they did not focus on clinical or technical aspects of treatment.
- Studies on syndromic craniosynostosis, as they focus on genetic or multi-suture involvement, which differs significantly from non-syndromic sagittal craniosynostosis.
- Research on non-sagittal craniosynostosis types (for example metopic, lambdoid, coronal, or combinations thereof), as these conditions require different diagnostic approaches and surgical techniques.
- Studies that focused on secondary craniosynostosis, such as those related to cranial deformities caused by external factors like shunt placement, were not considered relevant.
- Studies with small sample sizes, such as case reports with only 1–2 patients due to their potential lack of generalisability.

Finally, abstracts that only provided brief summaries of the condition without a detailed study description were also excluded.

## Results

### Literature search and study selection

An initial search using the search terms related to the operative technique yielded 160 records, the search related to the diagnostic tools yielded 141 records. In addition, 12 articles were found by further examining the reference lists of the originally identified publications.

**Fig. 1:**
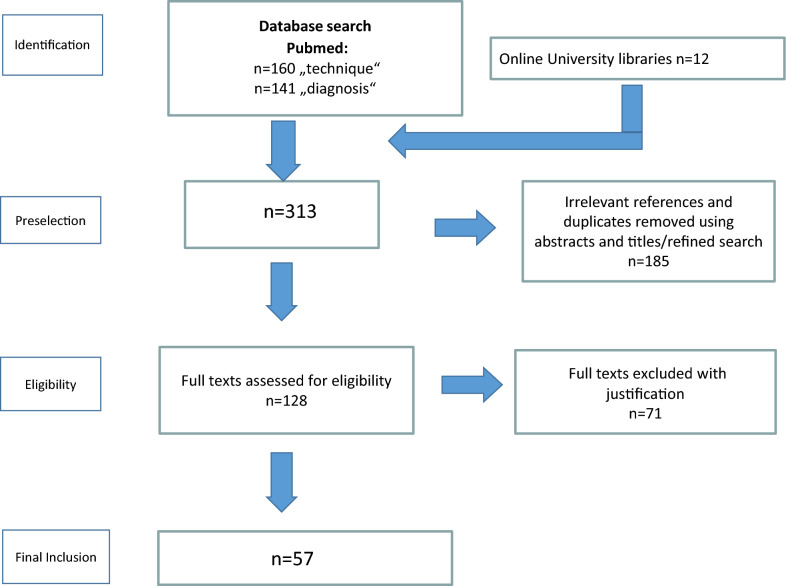
Flow chart of the study selection process

185 articles were removed as irrelevant to the stated research question or were duplicates. Of all 313 relevant publications, a total of 57 articles remained after the precise evaluation (Fig. 1).

The majority of publications were from the USA, followed by the Netherlands and United Kingdom. The extracted data included information on surgical procedures, diagnostic methods, patient-specific characteristics and outcomes.

After the literature had been completely reviewed, it was tabulated including the year of publication, first author, country of origin, type of study, diagnostic or surgical technique.

### Diagnosis technology

The diagnosis of craniosynostosis has been the subject of 19 studies, many of which yield overlapping findings that allow for a concise summary of key diagnostic methods (Table 1). This section highlights significant advancements and insights from pivotal studies, showcasing how these approaches have shaped current diagnostic strategies.

**Table 1:** Literature diagnostics of sagittal suture CSO

Year of publication	Title	First author	Country of origin	Type of study	Diagnostic technique
2003	Diagnosis and therapy of syndromic and non-syndromic craniosynostosis	Cornelia Cedzich	GER	Review article	X-Ray, CT, Scintigraphy
2006	Quantitative and qualitative assessment of morphology in sagittal synostosis: mid-sagittal vector analysis	Jeffrey R Marcus	CAN, USA	Retrospective study	CI, CT, MVSA
2006	Diagnosis of isolated sagittal synostosis: are radiographic studies necessary?	Deepak Agrawal	CAN	Retrospective study	Clinical examination, X-Ray, CT
2007	The diagnosis and treatment of single-sutural synostoses: are computed tomographic scans necessary?	Jeffrey A Fearon	USA	Prospective study	CT
2010	Associated (parallel) tomographic findings in patients with single-sutural synostosis	Renato da Silva Freitas	UK	Retrospective study	CT
2010	Prevalence and complications of single-gene and chromosomal disorders in craniosynostosis	Andrew O M Wilkie	UK	Retrospective cohort study	Chromosomal markers
2011	Actual concepts in scaphocephaly: an experience of 98 cases	Alexandru V Ciurea	RO	Retrospective study	Clinical examination, 3D CT
2012	Magnetic resonance imaging in isolated sagittal synostosis	Michael Engel	GER	Retrospective study	MRI
2014	"Black Bone" MRI: a potential alternative to CT with three-dimensional reconstruction of the craniofacial skeleton in the diagnosis of craniosynostosis	Karen A Eley	UK	Prospective observational study	“Black bone” MRI
2015	Diagnostic features of prematurely fused cranial sutures on plain skull X-rays	Tilmann Schweitzer	GER	Retrospective study	Plain skull X-Ray
2016	Cranial Ultrasound as a First-Line Imaging Examination for Craniosynostosis	Katya Rozovsky	CAN, ILS	Prospective observational study	CUS
2017	An Appraisal of the Cephalic Index in Sagittal Craniosynostosis, and the Unseen Third Dimension	Jeffrey A Fearon	USA	Retrospective study	CI
2017	Cranial ultrasound is a reliable first step imaging in children with suspected craniosynostosis	Laura Pogliani	ITA	Prospective study	CUS
2018	Prenatal ultrasound parameters in single suture craniosynostosis	Martijn J Cornelissen	NL	Retrospective study	Prenatal CI
2020	Combining deep learning with 3D stereophotogrammetry for craniosynostosis diagnosis	Guido De Jong	NL	Retrospective study	3D Stereography, deep learning algorithm
2021	Preoperative imaging patterns and intracranial findings in single suture craniosynostosis: a study from the Synostosis Research Group	Vijay M. Ravindra	USA	Retrospective study	Clinical examination, CT
2021	Updated Guideline on Treatment and Management of Craniosynostosis	Irene M J Mathijssen	NL	Guideline	US, 3D CT scan, skull X-ray, Black Bone MRI
2022	Prenatal Ultrasound Parameters of Twins with Sagittal Suture Craniosynostosis Question Mechanical Constraint as the Leading Cause	Kevin P Cinca	NL	Retrospective study	Prenatal US
2023	Modern treatment of craniosynostosis	Leon Schmidt	GER	Narrative review	Clinical examination, CUS

*X-Ray* Röntgen, *CT* computed tomography, *SPECT* scintigraphy, *MSVA* mid sagittal vector analysis, *CUS* cranial ultrasound, *CI* Cephalic Index, *BPD* biparietal diameter, *OFD* occipitofrontal diameter, *MRI* magnetic resonance imaging

In 2003, Cedzich and Farmand published the status of the necessary diagnosis of craniosynostosis. Accordingly, an existing suspected diagnosis should be confirmed at that time by a posterior-anterior and a lateral conventional X-Ray of the skull and a more detailed image should also be obtained by computed tomography (CT). A scintigraphy image (SPECT) of the occluded suture was also taken to be able to assess a possible final occlusion based on the basis of reduced activity. Sonographic imaging was only recommended for follow-up purposes [26].

The study by Agrawal et al. in 2006 analysed 114 children with isolated sagittal synostosis to assess if clinical diagnosis alone suffices for surgery. In most cases, the clinical findings were consistent with surgical and histopathological outcomes. Pathological examinations were performed in 104 of 114 children, all of which confirmed the diagnosis of sagittal craniosynostosis. Radiological investigations were often unnecessary, reducing radiation exposure and healthcare costs. The study concluded that surgery could proceed based on clinical diagnosis when symptoms are typical [27].

Marcus et al. introduced Mid-Sagittal Vector Analysis (MSVA) as a CT-based morphometric technique to assess cranial morphology in sagittal craniosynostosis in 2006. Pre- and postoperative CT scans of 16 patients were analysed, identifying three key affected regions: frontal, vertex, and occipital. MSVA effectively quantified preoperative deformity and postoperative correction. Uniquely, this method provided an objective, region-specific, and quantifiable assessment of cranial shape changes, offering a more precise evaluation of surgical outcomes compared to traditional qualitative assessments [28].

In their study of 67 patients with isolated craniosynostosis, Fearon et al. in 2007 demonstrated that CT imaging is unnecessary due to the condition’s clear clinical detectability and the associated risk of radiation exposure. In 66 of 67 children, the findings of the clinical and radiological examinations were similar in their description and associated diagnosis. Only one child with suspected lambdoid synostosis was radiologically diagnosed with positional plagiocephalus. In addition, 3/4 of the craniofacial surgeons rated CT as unnecessary for surgical intervention. Fearon et al. advise that due to the associated risks, including the need for additional sedation in children, and the significant cost of this procedure, CT scans should be reserved for cases where craniosynostosis cannot be clearly diagnosed by clinical assessment [29].

Wilkie et al. (2010) analysed 326 children with craniosynostosis (144 non-syndromic, 44.5% sagittal synostosis) who underwent genetic testing. Results showed that 21% of all non-syndromic had a genetic cause, mainly single-gene mutations (86%) and fewer chromosomal abnormalities (14%) with the FGFR3 P250R mutation being the most common. Coronal synostosis had the highest genetic association. In contrast, sagittal craniosynostosis showed minimal genetic links, with only a small percentage linked to single-gene mutations and even fewer chromosomal abnormalities. This suggests non-genetic factors play a greater role in sagittal craniosynostosis, though further research is needed. It provides valuable insights into surgical prognosis, recurrence risk, and informs the development of future surgical strategies [30].

In 2011 Ciurea et al. described the current state of diagnostics and therapy and emphasized the 3D CT scan as the preferred diagnostic method for the detection of scaphocephaly. Due to the radiation exposure caused by conservative X-ray diagnostics, this procedure was not recommended. Clinical examination methods such as head circumference or sonography were indicated as being of inferior importance [31].

As a pioneering new alternative to conventional CT imaging, Eley et al. (2014) examined the so-called “black bone MRI” (in 2D and 3D). A total of 13 children suffering from craniosynostosis underwent this new examination method and the results were compared with standard CT scans. Within a “black bone image”, physiological sutures contrast from the cranial bones with increased signal intensity, prematurely closed ones do not, so the clinical potential for diagnosis without radiation became evident [32]. Classic MRI was still recommended if there is clinical evidence of an intracranial anomaly [3, 4, 33].

In 2015, Rozovsky et al. investigated the utility of cranial ultrasound (CUS) as a first-line imaging modality for diagnosing craniosynostosis in infants under 12 months, benchmarking its diagnostic accuracy against radiography. Their study revealed complete concordance between CUS and radiography in assessing especially the sagittal sutures. Overall, the results demonstrated that CUS possesses high sensitivity (100%) and specificity (98%), reliably detecting suture abnormalities while eliminating the risks associated with ionising radiation. These findings underscore the value of CUS as a safer, non-invasive alternative to radiography, particularly for initial assessments [34, 35].

Fearon et al. examined in 2017 a total of 392 craniosynostosis patients to validate the CI method, which is calculated by multiplying the maximum skull width by 100 and dividing it by the skull length. They found no significant correlation between the measured CI and the subjectively assessed severity. In addition, they note that the CI method cannot capture certain postoperative changes, such as changes in occipital skull height after sagittal suture synostosis surgery, or accurately reflect the complexity of skull deformities [7].

Cornelissen et al. examined in 2018 whether prenatal ultrasound can help detect sagittal craniosynostosis scaphocephaly among others. Researchers analysed 20-week ultrasounds from 41 affected foetuses and 82 controls, focusing on skull measurements like biparietal diameter (BPD), occipitofrontal diameter (OFD), and the CI. Findings showed that foetuses with scaphocephaly had a significantly lower CI (0.76 vs. 0.79; p = 0.000), but CI alone was not reliable for screening at 20 weeks. However, a growth deviation in BPD from week 20 onward suggested that 3D imaging of cranial sutures may be beneficial in suspected cases [36].

In 2020 De Jong et al. investigated the use of 3D stereography as an alternative diagnostic method to CT examinations, as well as to supplement a possible lack of expertise. By capturing photographic images of the child's head from different angles and merging them using special software, a precise three-dimensional individual model is created. In combination with a “deep learning algorithm”, this enabled the visualisation of deviations from the ideal form, whereby a comparison with a healthy cohort is applied. The accuracy of this method is clearly demonstrated by the exact classification of 195/196 cases (99.5%), with a sensitivity of 100% and a specificity of 96% for scaphocephaly [8].

In 2021 Ravindra et al. investigates the necessity of preoperative CT imaging in infants with yet clinically diagnosed single suture craniosynostosis, including sagittal craniosynostosis. The authors analysed whether CT findings confirmed the clinical diagnosis and influenced surgical decision-making. Results demonstrated that in most of the cases, CT imaging corroborated the clinical diagnosis and rarely altered the surgical approach. Consequently, the authors suggest that routine preoperative CT imaging may not be essential in cases with a clear clinical diagnosis, thereby potentially reducing radiation exposure in affected infants [37].

The study by Cinca et al. published in 2022, explores the potential role of mechanical constraints in the development of sagittal suture craniosynostosis in twin pregnancies. The study analyses prenatal ultrasound data and suggests that restricted space in the uterus could contribute to the early fusion of the sagittal suture, leading to an abnormal head shape. The authors propose that mechanical factors, such as crowding or abnormal positioning of the twins, may be a significant factor in the development of SSC, alongside genetic causes [38].

In the article published in 2023, Schmidt et al. explained that in sonography an echo-poor gap between the adjoining cranial bones can be recognised in an intact suture, which corresponds to the fibrous unclosed suture; in an ossified cranial suture, however, a corresponding gap is missing [2]. The sensitivity of this method is reported to be almost 100%, the specificity is 86–98% compared to the CT collective [2].

In addition to sonography, Schmidt et al. recommend in 2023 other, in some cases very classic, minimally invasive methods for confirming a possible suspected diagnosis of craniosynostosis during an initial clinical examination. The pathognomonic shape of the skull, viewed from the vertex, is usually the first thing that attracts attention. The sutures can then be palpated; a hardened (osseous) palpable thickening would be noticeable. Another recommended method of assessment would be to determine the CI. This parameter is particularly appropriate for the presence of scaphocephaly and for follow-up assessment, even after surgery [2].

### Surgical perspectives

38 studies have investigated surgical therapies for craniosynostosis, with many reporting overlapping findings. These studies largely agree on the efficacy and limitations of various techniques, highlighting the importance of age, method, and postoperative care in achieving optimal outcomes. Below, key studies and their findings are summarised to provide an overview of therapeutic advancements (Table 2).

**Table 2:** Surgical techniques

Year of publication	Title	First author	Country of origin	Type of study	Therapy technique
1997	Surgical management of sagittal synostosis: a comparative analysis of strip craniectomy and calvarial vault remodeling	Todd A. Maugans	USA	Retrospective study	SC, CVR
1998	Endoscopic craniectomy for early surgical correction of sagittal craniosynostosis	David F Jimenez	USA	Prospective study	ESC, barrel stave, helmet therapy
1999	Sagittal craniosynostosis outcome assessment for two methods and timings of intervention	Jayesh Panchal	USA	Retrospective study	SC, SCT
1999	Endoscopic craniectomy for early correction of craniosynostosis	Constance M Barone	USA	Retrospective study	ESC, Marchac technique
2001	Outcome analysis for correction of single suture craniosynostosis using resorbable fixation	Albert MD Losken	USA	Retrospective study	Resorbable plating system
2001	Clinical outcome of the modified pi-plasty procedure for sagittal synostosis	Guimarães-Ferreira, José M	SWE	Retrospective study	Modified Pi-plasty procedure
2004	Endoscopy-assisted wide-vertex craniectomy, barrel stave osteotomies, and postoperative helmet molding therapy in the management of sagittal suture craniosynostosis	David F Jimenez	USA	Prospective study	ESC + barrel stave + helmet therapy
2009	Single sutural craniosynostoses: surgical outcomes and long-term growth	James A Fearon	USA	Retrospective study	CVR
2009	Spring-assisted surgery-a surgeon's manual for the manufacture and utilization of springs in craniofacial surgery	Jeremy Pyle	USA	Retrospective study	SAC
2011	Endoscopically assisted versus open repair of sagittal craniosynostosis: the St. Louis Children's Hospital experience	Manish N. Shah	USA	Prospective study	SC, CVR
2012	Endoscopic technique for sagittal synostosis	David F Jimenez	USA	Retrospective study	ESC
2012	Minimally invasive strip craniectomy for sagittal synostosis	Barbu Gociman	CAN	Retrospective study	ESC
2013	Results of early surgery for sagittal suture synostosis: long-term follow-up and the occurrence of raised intracranial pressure	Marie-Lise C. Van Veelen	NL	Retrospective study	SC
2013	Total cranial vault remodeling for isolated sagittal synostosis: part I Postoperative cranial suture patency	Mitchel Seruya	AUS	Retrospective study	CVR
2015	Frontobiparietal remodeling with or without a widening bridge for sagittal synostosis: comparison of 2 cohorts for aesthetic and functional outcome	Marie-Lise C Van Veelen	NL	Retrospective study	FBR, mFBR
2015	Safety of Open Cranial Vault Surgery for Single-Suture Craniosynostosis: A Case for the Multidisciplinary Team	Craig B Birgfeld	USA	Retrospective study	CVR
2016	Transsutural distraction osteogenesis for 285 children with craniosynostosis: a single-institution experience	Dong Ha Park	KOR	Retrospective cohort study	Transsutural distraction osteogenesis (TSDO)
2016	Triple square extended osteotomies for treatment of scaphocephaly (Renier's "H" technique modification)	Mirko Micovic	SRB	Retrospective analysis / case series	Triple square extended osteotomies
2017	Spring-Assisted Cranioplasty for the Correction of Nonsyndromic Scaphocephaly: A Quantitative Analysis of 100 Consecutive Cases	Will Rodgers	UK	Retrospective cohort study	SAC
2017	Surgical Treatment of Nonsyndromic Craniosynostosis	Kristen A Klement	USA	Retrospective study	Foreshortening and Lateral Expansion of the Cranium Activated by Gravity
2018	Twenty-Year Outcome Experience with Open Craniosynostosis Repairs: An Analysis of Reoperation and Complication Rates	Kerry A Morrison	USA	Retrospective study	CVR
2019	Osteoclastic craniectomy for scaphocephaly in infants results in physiological head shapes	Markus Lehner	GER, CH	Single center observational study	Osteoclastic craniectomy
2019	A comparison of endoscopic strip craniectomy and pi craniectomy for treatment of sagittal craniosynostosis	Suresh N Magge	USA	Retrospective Study	Pi craniectomy vs. endoscopic strip craniectomy
2020	Minimally Invasive Endoscopic Surgery for Infantile Craniosynostosis: A Longitudinal Cohort Study	Coleman P Riordan	USA	Longitudinal cohort study	ESC
2020	Three-Dimensional Calvarial Growth in Spring-Assisted Cranioplasty for Correction of Sagittal Synostosis	Naiara Rodriguez-Florez	UK	Clinical study	SAC
2021	Spring-mediated cranioplasty versus endoscopic strip craniectomy for sagittal craniosynostosis	Shih-Shan Lang	USA	Retrospective study	ESC, SMC
2021	Partial suturectomy for phenotypical craniosynostosis caused by incomplete fusion of cranial sutures: a novel surgical solution	David C Lobb	USA	Retrospective study	Partial suturectomy
2021	Single incision endoscopic strip craniectomy for sagittal craniosynostosis	Edward S Ahn	USA	Video article	Single incision SC
2021	Ultra-early synostectomy and cranial remodeling orthoses in the management of craniosynostoses	Aaron Mohanty	USA	Retrospective study	Ultra early synostectomy
2021	Single Segment Neo-Bandeau Fronto-Orbital Advancement in Children With Craniosynostosis: Technique Adaptation and Craniometric Analysis	Zachary D Zapatero	USA	Retrospective study	Fronto-Orbital-Advancement
2022	Endoscopic treatment of sagittal suture synostosis—a critical analysis of current management strategies	Verena Fassl	GER	Systematic review	Endoscopic techniques
2022	Single sagittal craniosynostosis surgical treatment with the "Peau d́ours" technique. Single-center experience in Mexico	Jose Ascencion Arenas-Ruiz	MX	Retrospective study	"Peau d́ours" technique
2022	Flexible endoscope-assisted suture release and barrel stave osteotomy for the correction of sagittal synostosis	Jason Labuschagne	USA, UG	Retrospective study	FEASR
2022	Endoscopic strip craniectomy with molding helmet therapy versus spring-assisted cranioplasty for nonsyndromic single-suture sagittal craniosynostosis: a systematic review	Alexandra Valetopoulou	UK	Systematic review	ESC, SMC
2023	Technical evolution of pediatric neurosurgery: craniosynostosis from 1972 to 2023 and beyond	Federico Di Rocco	USA, FRA	Review article	Selection of minimally invasive decompression surgery to major reconstructive operations
2023	Evaluation of Helmeting Therapy Duration After Endoscopic Strip Craniectomy for Metopic and Sagittal Craniosynostosis	Huan T Nguyen	USA	Retrospective study	ESC, helmet therapy
2023	Comparison of head shape outcomes for three minimally invasive strip craniectomy techniques for sagittal craniosynostosis	Imran Rizvi	USA	Retrospective study	Minimal invasive SC
2024	Understanding the influence of surgical parameters on craniofacial surgery outcomes: a computational study	Kun-Huan He	UK	Retrospective study	SAC

*CVR* calvarial vault remodeling, *ESC* endoscopic strip craniectomy, *FBR* frontoparietal remodeling, *FEASR* flexible endoscope-assisted suture release, *mFBR* modified frontoparietal remodeling, *SAC* spring assisted cranioplasty, *SAT* subtotal calvariectomy, *SC* strip craniectomy, *SMC* spring mediated cranioplasty)

The Pi procedure, introduced by Jane et al. in 1978 and named after the Greek letter π, involves dynamic cranioplasty using osteotomies shaped like the symbol π to enable controlled cranial expansion [39]. Unlike total cranial vault remodeling, the procedure preserves the sagittal suture and reshapes the skull with bone strip removal and compression [39, 40]. Magge et al. noted no significant reduction in surgical burden compared to endoscopic craniectomy, though its long-term benefits remain debated [41]. In a 20-year retrospective analysis of open cranial vault repairs, Morrison et al. focused on reoperation and complication rates, emphasising that despite the historical importance of open techniques like these, they do not necessarily reduce complications or the need for additional surgeries [42]. Their studies underscore the ongoing challenges in achieving optimal long-term outcomes and the importance of evaluating reoperation rates and exploring alternative approaches.

In the early 1990s, Maugans et al. compared the strip craniectomy (SC) method with the more extensive calvarial vault remodeling (CVR) procedure for the treatment of sagittal craniosynostosis to delay premature ossification. CVR is an invasive surgical technique for treating craniosynostosis by removing, reshaping, and repositioning parts of the skull. This procedure corrects skull deformities, allows for normal brain growth, and helps prevent increased intracranial pressure. In contrast to the CVR method, bone defects were found in 59% of the SC patients at the final examination, and two patients had to be operated once again due to suboptimal cosmetic results (with the CVR method). The results showed that the effectiveness of SC procedures decreases with increasing age, in contrast to the CVR method [43].

More studies on CVR for craniosynostosis, including work by Birgfeld et al. and Seruya et al., focus on surgical safety, outcomes, and suture patency. Birgfeld et al. emphasize the role of multidisciplinary teams in ensuring safe open cranial vault surgery. Seruya et al. retrospectively assessed cranial suture patency using postoperative CT scans taken approximately 6–12 months after total cranial vault remodeling for isolated sagittal synostosis. A neurosurgical consultant rated the bilateral coronal and lambdoid sutures based on axial and 3D-reconstructed imaging, assigning scores of 0 (closed), 1 (partially open), or 2 (fully open). Partial patency was defined by alternating open and fused segments across consecutive axial slices. Scores from the four sutures were summed to yield a total score between 0 and 8. Only repositioned vault regions were evaluated, with the basilar skull excluded. This structured approach enabled consistent, quantifiable comparisons across patients and subgroups. Both highlight the effectiveness of CVR but stress the importance of surgical planning and postoperative monitoring [44, 45].

Panchal et al. (1999), examined 40 infants with sagittal craniosynostosis to determine whether the postoperative outcome in relation to the cranial index can be linked to the child's age and the extent of the operation. He concluded that, extended SC for sagittal craniosynostosis does not lead to a normal ratio between skull width and length, even if performed before 4 months of age. Nevertheless, performing subtotal calvariectomy (SCT) within the first 13 months of life typically restores a normal skull width-to-length ratio in most children [46].

Due to the persistently existing complication and revision rates of open surgical approaches, Jimenez & Barone developed an alternative method for treating craniosynostosis in 1998, based on four successfully performed operations. In addition to the removal of the premature suture, this technique also included the so-called Barrel-Staves osteotomies of the temporal and parietal bones [9]. Barrel-stave osteotomies are a surgical method to reshape bones, often used in craniosynostosis treatment. Multiple parallel bone cuts increase flexibility, allowing the bone to be reshaped and stabilised with plates or screws, ensuring structural integrity [47, 48]. They also used helmet therapy afterwards, initially for sagittal suture synostosis [1, 49]. An average operating time of 1.68 h with an average blood loss of 54.2 ml was determined. Three out of four patients did not require a blood transfusion and could be discharged after just 24 h. In a subsequent follow-up visit after eight to 15 months, the authors found that all patients had undergone successful and sustained correction of the scaphocephaly [9].

In their 2004 publication, the authors further explored endoscopically assisted wide vertex craniectomies combined with bitemporal and biparietal Barrel-Staves osteotomies for the treatment of 139 patients with sagittal synostosis. The patients were on average 3.6 months old. A total of 9% (two intraoperative, 12 postoperative) required blood transfusions, the mean blood loss was 29 ml. 120 out of 139 patients could be discharged on the following day. The results showed that 87% of patients had excellent outcomes with a cephalic index of over 75, 8.7% had good outcomes between 70 and 75 and 4.3% had poor outcomes (< 70) had poor outcomes. The mortality rate was lower than with traditional reconstruction methods such as full CVR, and no serious complications occurred [15].

One year later this group presented their 16 years of experience to date in treating a total of 256 patients with sagittal synostosis using the method described above. Blood loss was reduced to an average of 27 ml and the transfusion rate to 7%. Discharge was also possible after an average of one day. The very high success rate of 87% remained similar to subsequent years despite the change in CI guidelines (87% of patients achieved a CI above 80, 9% a CI of 80–70 as good and 4% < 70 as poor) [50].

Recent studies on endoscopic strip craniectomy (ESC) for craniosynostosis, including work by Lobb et al., Valetopoulou et al., Ahn and Bhandarkar and Magge et al. explore various aspects of the technique [41, 51–54]. Common themes include its effectiveness in treating sagittal and metopic synostosis, the use of helmet therapy post-surgery, and comparisons with other surgical approaches like spring-assisted cranioplasty. Differences include the focus on patient age, incision techniques, and long-term outcomes [41, 51–53]. Further contributing to this field, Riordan et al. conducted a longitudinal cohort study examining ESC for infantile craniosynostosis, demonstrating favourable outcomes for infants undergoing ESC [55]. Similarly, Gociman et al. explored minimally invasive strip craniectomy for sagittal synostosis in a retrospective study, providing evidence for the technique's efficacy in treating this condition [56]. These studies, along with others, underscore the growing body of evidence supporting the use of ESC as a treatment for craniosynostosis, especially in younger patients.

Fearon et al. 2009 conducted in 2009 a retrospective analysis of children with nonsyndromic single sutural synostosis who underwent a single CVR procedure. The study found that while cranial indices normalised with low preoperative rates and minimal complications, post-surgical growth did not fully return to normal, with a tendency for the calvaria to revert towards the original deformity. The authors concluded that surgeons should aim for overcorrection rather than just normalisation of the skull shape, particularly in younger patients. This raised concerns about the effectiveness of endoscope-assisted procedures and postoperative molding. Furthermore, delaying surgery may improve long-term aesthetic outcomes, but the timing should carefully consider potential brain development risks and the need for full reconstruction in children over 10 months of age [57].

In 2011, Sha et al. investigated differences in the efficacy and morbidity of minimally invasive endoscopic wide vertex SC with the use of barrel-starves osteotomies and postoperative helmet orthosis for an average of 8.7 months compared to open cranial vault reconstruction for sagittal craniosynostosis. A total of 89 children were included, 47 underwent endoscopic surgery at an average age of 3.6 months and 42 underwent open surgery at an average age of 6.8 months. The endoscopic method not only showed a significantly lower mean blood loss (29 ml compared to 218 ml in the open method) and a lower transfusion rate (all openly operated children received one, whereas only 3 endoscopically operated children received one), but also allowed a significantly shorter postoperative hospitalisation (1.2 days compared to 3.9 days in open procedures). It is also noteworthy that the pre- and postoperative CI after 13 months were comparable in the endoscopically treated children (68% and 76%), while the open procedures had comparable CI values after 25 months (68% and 77%) [58].

In 2013, Van Veelen et al. investigated the outcome of extended SC on a total of 79 consecutive sagittal suture synostosis patients. Four OP—techniques were used: A–D, from simple bilateral parietal flap with breaking out the bone roof to remodeling the parietal flap by adding triangular incisions and bending or suturing. Compared to the initial CI, variant D, in which the excised sagittal strip is fixed rotationally between the parietal lobes, showed the greatest initial improvement. After two years of follow-up, however, no significant difference was found between the variants. The mean blood loss was 230 ml, and four patients had to undergo further surgery due to increased intracranial pressure [59].

In 2015, Van Veelen et al. compared the classic fronto-parietal reconstruction with a modified version of this technique, in which the removed bone piece is rotated by 90° and fixed between the parietal bones to increase the width of the skull. The study included 69 children diagnosed with sagittal suture synostosis. During the follow-up the head circumference decreased in both groups, whereby the preoperative head circumference had the decisive influence, but not the chosen surgical technique. Furthermore, aesthetic results and associated complications like the prevalence of headaches were found to be comparable in both groups. Both groups also showed a similar blood loss of 1174 ml in the classic group and 914 ml in the modified group. Van Veelen et al. concluded that the addition of a widening bridge in the context of late complete remodeling (older than 9 months) significantly and long-lastingly improves CI [60].

In a prospective multicenter registry study conducted by Lang et al. 2021, which included children between 2012 and 2019, the results of minimally invasive surgical methods, ESC versus spring-mediated cranioplasty (SMC) were compared. The study involved a total of 676 children, all under the age of 6 months, who were diagnosed with sagittal suture synostosis. Among them, 580 were ESC infants from 32 centres, and 96 were SMC infants from five centres. The results indicated no significant difference in the incidence of a transfusion-free hospital course between the two groups. However, the likelihood of being admitted to the intensive care unit, as well as the length of stay and the overall hospitalisation duration, was greater in the spring-assisted surgery group, potentially due to the hospital's protocol [61]. Further supporting the efficacy of SMC, Rodriguez-Florez et al. demonstrated significant three-dimensional calvarial growth following this intervention, highlighting its potential in reshaping cranial morphology [62]. Similarly, Rodgers et al. analysed 100 consecutive cases of nonsyndromic scaphocephaly treated with SMC and confirmed positive outcomes, reinforcing its role as a viable alternative to ESC [63]. Additionally, Pyle provided a comprehensive surgical manual detailing the manufacturing and application of springs in craniofacial surgery, further contributing to the standardisation of this technique [64].

## Discussion

This literature review demonstrates that there have been significant developments in the diagnosis of craniosynostosis over the last 30 years because of technological progress. In the past three decades, diagnosis was mainly carried out using X-rays or CT scans, but this was problematic due to the radiation exposure for the developing brain [65]. For example, Cedzich and Farmand 2003 recommended a combination of conventional radiographs and CT imaging for diagnosis [26], while Fearon et al. 2007 advised against unnecessary CT images due to radiation risks and the good clinical accessibility especially of CI [29]. Brenner et al. concluded already in 2001 that paediatric CT scans significantly increase the lifetime risk of cancer mortality due to the higher radiation sensitivity of children and cited the urgent need to reduce CT radiation doses in paediatrics to minimise these risks while maintaining diagnostic efficacy [66]. Studies by Pearce et al. [67] and Sheppard et al. [68] showed the increased risk of leukaemia and brain tumuors from CT applications with a cumulative dose of 50–60 mGy.

In 2010 da Silva Freitas et al. emphasises the value of preoperative CT scans in improving diagnostic accuracy for sagittal craniosynostosis and associated conditions. While sagittal synostosis is often identified clinically, CT helps detect additional suture involvement, such as combined sagittal and metopic synostosis, which may go unnoticed without imaging. This ensures a more comprehensive surgical plan (for example special operational positioning) and optimal cranial reshaping. CT also aids in identifying brain abnormalities (for example subarachnoid cysts, cerebral atrophy) and noncerebral conditions, facilitating timely referrals and interventions. Overall, low-dose paediatric CT persists valuable for accurate diagnosis and comprehensive presurgical planning [69].

However, the advancement of diagnostic strategies has increasingly highlighted the importance of alternative imaging modalities that eliminate radiation exposure. For example, Eley et al. examined the “black bone MRI”, which enables precise cranial suture assessment without radiation but necessitates anaesthesia in young children [5, 32, 33], while sonography up to 13 months of age can distinguish an open from a closed suture with a reported sensitivity of 100% and specificity of 86–98% [2, 32], without any necessary anaesthesia. The benefits of sonography are its rapid availability and the lack of radiation. The disadvantages are that the findings are dependent on the diagnostic physician and are difficult to interpret in older children due to the fact that some of the sutures are already physiologically closed or the disease may be at an already advanced stage [34, 35].

De Jong et al. presented a radiation-free alternative that combines 3D stereophotogrammetry with a deep learning algorithm to assess deviations in skull shape. Using standardised photographic data, this method enables the accurate reconstruction of three-dimensional surfaces and supports both diagnosis and long-term monitoring [8, 31]. Modern AI models use defined anatomical landmarks, such as the tragus or the outer corner of the eye, extracted from datasets of healthy individuals or corpses, to create normative references [70]. Patient-specific skull measurements are then compared to these standards across multiple axes and angles (e.g. interaural distance and head circumference), enabling the objective identification of morphological deviations [70]. These quantitative data can be evaluated both longitudinally, for pre- and postoperative comparisons, and in relation to population norms, for benchmarking purposes. Additionally, newer approaches combine these morphometric results with patient-reported outcomes, providing a more comprehensive evaluation of treatment success [70]. Together with the integration of artificial intelligence into image analysis, photogrammetry enables a reliable, radiation-free assessment of skull morphology comparable to the results of a CT scan [71]. Integrating artificial intelligence into image analysis improves disease detection and supports advanced surgical planning and any necessary postoperative helmet treatment [8]. However, measurement reliability can be compromised by different imaging techniques and inconsistent landmark placement, leading to uncertainty in the measurements [70].

The effective use of these technologies still requires specialized expertise for accurate interpretation and further appropriate treatment recommendations based on the latest research findings [2, 6]. This is accompanied by several practical limitations in clinical, like the considerable cost of equipment and the need for patient compliance during image acquisition- particularly challenging in very young children who may be unable to remain still [70]. Image quality can also be compromised by artefacts, for example caused by hair when protective caps are not used [70]. Moreover, the reliable identification of anatomical landmarks may be difficult in cases of abnormal cranial morphology [70]. Finally, the wide range of available scanning systems and algorithms, in the absence of robust comparative studies, presents a barrier to standardisation and widespread clinical acceptance [72].

While imaging plays a crucial role in craniosynostosis diagnosis, several studies underscore the continued relevance of thorough clinical examination, providing a reliable and cost-effective alternative to imaging. Both Agrawal et al. and Schmidt et al. highlighted that clinical evaluation alone can accurately identify the condition, eliminating the need for imaging and reducing associated risks. Early diagnosis through a detailed physical examination not only enables timely surgical intervention but also helps minimise healthcare costs, underscoring the value of clinical expertise in managing this condition [2, 27].

Fearon et al validated the CI, as part of a clinical examination, and found that it does not always represent the severity of craniosynostosis, as it now categorises a three-dimensional malformation using a two-dimensional indication or measure [7]. Marcus et al. have emphasised already in 2006 in their study that the CI is a relative measure that cannot differentiate between frontal and occipital bossing or quantify deformity severity. For example, complete sagittal suture involvement causes both frontal and occipital bossing, while partial involvement affects only the front or back of the skull [28].

Prenatal detection of sagittal craniosynostosis remains an area of growing interest. Cornelissen et al. and other authors highlighted the potential of prenatal ultrasound, with BPD deviations suggesting improved accuracy using 3D imaging. While ultrasound is non-invasive and widely available, CI alone is unreliable, and the feasibility of advanced imaging in routine screening requires further study [36, 38].

The role of genetic factors in craniosynostosis etiology varies by subtype. Wilkie et al. showed that sagittal craniosynostosis has minimal genetic links, reducing recurrence risk but complicating early diagnosis. Unlike coronal synostosis, which has stronger genetic associations, sagittal cases likely involve environmental factors. Understanding these influences could improve risk assessment and surgical planning [30]. More recently Cinca et al. proposed that mechanical constraints in twin pregnancies may contribute to sagittal suture fusion, highlighting the multifactorial nature of this condition [38].

The Pi procedure, first introduced by Jane et al. in 1978, was one of the earliest surgical approaches developed for the treatment of craniosynostosis. By enabling controlled cranial expansion through π-shaped osteotomies while preserving the sagittal suture, it represented a pioneering attempt to address the underlying pathophysiology of the condition beyond simple suturectomy [39, 40]. While pioneering at that time, its long-term efficacy remains debated. Magge et al. found no significant reduction in surgical burden compared to ESC, and Morrison et al. reported that traditional open vault repairs do not necessarily lower complication rates or the need for reoperation [41, 42]. Additionally, Fearon et al. emphasised the importance of growth prognosis in open cranial remodeling outcomes, while Shah et al. compared endoscopic and open techniques, and found endoscopic approaches result in significantly lower transfusion rate, shorter hospital stays and comparable postoperative CI, suggesting reduced morbidity with similar outcomes to open surgery [57, 58].

The surgical management of sagittal synostosis has since evolved significantly, shifting from early procedures like the Pi technique towards more comprehensive approaches such as CVR and SC [1, 11].

Initially, SC focused on releasing the fused sagittal suture but did little to correct skull shape abnormalities, such as frontal bossing or occipital elongation [14]. Due to high revision rates, caused for example, by renewed ossification, new procedures became necessary [73]. In the 1990s, the comparison of SC with CVR by Maugans et al. made it clear that, despite the more extensive procedure with significantly increased blood loss in contrast to SC, immediate and long-term correction is possible, especially in older patients with more pronounced findings [43]. This was further substantiated by Panchal et al. in 1999, who found that extended SC did not result in normal skull proportions, even when performed before four months of age, subtotal calvariectomy (SCT) could result in normal skull dimensions if conducted before 13 months [46]. In a more recent comparative study, Birgfeld et al. and Seruya et al. emphasised that multidisciplinary surgical planning is crucial in ensuring both the safety and effectiveness of CVR, underscoring the importance of postoperative monitoring to optimise long-term outcomes [44, 45].

Blood loss remains a critical factor in evaluating surgical methods. Van Veelen et al. demonstrated that modified frontoparietal remodeling (mFBR) by rotating a bone segment to widen the skull significantly reduced the blood loss (914 ml in the mFBR group vs. 1174 ml in the FBR group) while sustaining long-term CI improvements [60]. In contrast, the introduction of ESC with barrel staves in the late 1990s by Jimenez and Barone, which was made possible by technological advances in image-guided surgery, instrumentation and neuroendoscopy, resulted in a reduction in blood loss to an average of 27 ml, markedly decreasing transfusion rates and mortality risk [9, 50]. In addition to the reduced blood loss, after 16 years of using this method, patients experienced fewer transfusion and shorter hospital stays compared to those who underwent open procedures [15, 50].

The integration of helmet therapy after endoscopic procedures showed an increased corrective effect, especially for the sagittal Craniosynostosis [1, 53, 55, 56]. However, it should be noted at this point that due to the advanced age of open surgical approaches and the more complex findings, potentially more difficult extensive operations must be performed compared to younger children who may be operated on with less pronounced findings [12].

The use of springs to treat prematurely fused sagittal suture, especially in cases where families refuse helmet therapy or the child is too old for it, allows two-dimensional expansion of the skull and has been shown to be a successful addition and minimally invasive alternative [51, 64]. The results of SMC compared with SC showed comparable transfusion-free hospital courses, but a longer hospitalisation period was observed. In addition, they must be removed in a separate procedure once the treatment goal has been achieved [61, 74, 75]. Rodriguez-Florez et al. demonstrated that spring-assisted cranioplasty for sagittal synostosis facilitates three-dimensional calvarial growth, leading to improved cranial shape and symmetry over time [62]. In contrast, Rodgers et al. reported that while the technique yielded modest CI improvement, it required a secondary procedure for spring removal [61, 74, 75] and carried a risk of complications, including cerebrospinal fluid leaks and reoperations, in a cohort of 100 cases [63].

Additionally, Riordan et al. and Gociman et al. supported the efficacy of minimally invasive techniques such as ESC, particularly in younger patients, reinforcing their role as viable alternatives in the surgical management of craniosynostosis [55, 56]. Mohanty et al. further highlighted the benefits of ultra-early synostectomy in combination with helmet therapy, emphasising its potential to optimise cranial remodeling outcomes [76].

In addition to finding a diagnosis with the 3D stereography method described from de Jong et al. 2020, this method also enables an effective follow-up assessment, and the data collected can also be used for the post-operative production of a personalised helmet orthosis [77–79].

Concerns about metallic plates in paediatric craniofacial surgery, such as growth restrictions and long-term effects, have led to the use of resorbable plating systems. These systems provide effective stabilisation with no loss in rigidity and improve surgical outcomes. Reoperation rates for resorbable plates in single-suture synostosis were comparable to traditional titanium plates. Resorbable plates have become the preferred choice, offering a safer, effective alternative without the complications of permanent implants and are now the standard for infant cranial reconstruction [80].

Despite ongoing advancements, several alternative surgical techniques remain limited to specialized centres. Approaches such as the Peau d'Ours technique, triple square extended osteotomies, and transsutural distraction osteogenesis have been explored but lack widespread clinical acceptance due to their complexity and the availability of more established techniques [54, 81–84]. Although these techniques have shown promise, their efficacy and safety require further validation through long-term studies before they can be recommended as standard treatments.

Similarly, Labuschagne et al. demonstrated that flexible endoscope-assisted suture release combined with barrel stave osteotomies represents another minimally invasive alternative for sagittal synostosis correction [85]. Furthermore, Klement et al. introduced an innovative cranial expansion technique activated by gravitational forces, offering a novel approach that may warrant further investigation [86]. In addition, Zapatero developed an adapted fronto-orbital advancement technique aimed at optimizing skull contour, further contributing to the refinement of surgical strategies in craniosynostosis management [87].

Early diagnosis enables appropriate planning and intervention, also to support the normal growth of the skull and brain in the best possible way, but also as the condition can be treated minimally invasively in the first months of life with reduced mortality [15, 88]. Nevertheless, paediatricians should improve their skills in the assessment of cranial anomalies through regular training and further education and thus help to prevent potentially serious complications in affected children. This method also requires specialist knowledge, as treatment recommendations should be discussed on the basis of the latest evidence-based research and literature [89].

## Conclusion

Today the most common approach for evaluating suspected craniosynostosis is a detailed clinical examination by skilled professionals, where characteristic skull morphology serves as the primary indicator for provisional diagnosis. Diagnostic confirmation typically involves the use of methods such as measurement of the head circumference, ultrasonography and if required and technically possible three-dimensional stereophotography. In more challenging or atypical cases, additional imaging techniques like CT and MRI may be employed [2, 7, 8, 37]. Two primary surgical approaches are utilized for the treatment of craniosynostosis: the (flexible) endoscopic-assisted technique and the conventional open surgical approach, with the choice depending on individual surgical planning and case specifics. Endoscopic approaches are preferred in infants younger than 6 months, while open surgical techniques are typically used in older children [2, 50, 85]. The endoscopic-assisted method offers advantages such as reduced blood transfusion rates, shorter surgical times, less scarring, and shorter hospital stays. However, it often necessitates postoperative helmet therapy to guide skull remodeling [9, 10]. In contrast, open surgery provides immediate correction of skull shape but is associated with significant drawbacks, including greater invasiveness, increased blood loss (a common intra- and postoperative complication), larger bone defects, and more pronounced scarring [10, 11].

Over the last 30 years, there has been considerable development in the diagnosis, surgical techniques and treatment of craniosynostosis due to technological progress. Non-invasive approaches for influencing cranial fusion could represent promising alternatives to major open surgical interventions. Pharmacological interventions that specifically target the signalling pathways involved could pave the way for gentler and more effective treatment options [90]. The coming years will therefore be groundbreaking in terms of further optimising the diagnosis and treatment of sagittal suture craniosynostosis, reducing mortality rates and improving patients' quality of life.

## Data Availability

Not applicable.

## Abbreviations

CI: Cephalic Index
CSO: Craniosynostosis
CT: Computed tomography
CUS: Cranial ultrasound
CVR: Calvarial vault remodeling
ESC: Endoscopic strip craniectomy
FBR: Frontoparietal remodeling
FEASR: Flexible endoscope-assisted suture release
mFBR: Modified frontoparietal remodeling
MRI: Magnetic resonance imaging
MVSA: Mid-sagittal vector analysis
SAC: Spring assisted cranioplasty
SAT: Subtotal calvariectomy
SC: Strip craniectomy
SMC: Spring mediated cranioplasty
X-Ray: Radiography
